# Capillary Electrophoresis Mass Spectrometry-Based Metabolomics of Plasma Samples from Healthy Subjects in a Cross-Sectional Japanese Population Study

**DOI:** 10.3390/metabo11050314

**Published:** 2021-05-13

**Authors:** Hiroyuki Yamamoto, Makoto Suzuki, Rira Matsuta, Kazunori Sasaki, Moon-Il Kang, Kenjiro Kami, Yota Tatara, Ken Itoh, Shigeyuki Nakaji

**Affiliations:** 1 Human Metabolome Technologies, Inc., 246-2 Mizukami, Kakuganji, Tsuruoka, Yamagata 997-0052, Japan; makosu0917@gmail.com (M.S.); rira.matsuta@humanmetabolome.com (R.M.); sasaki@humanmetabolome.com (K.S.); mikang@humanmetabolome.com (M.-I.K.); kkami@humanmetabolome.com (K.K.); 2 Department of Metabolomics Innovation, Hirosaki University Graduate School of Medicine, 5 Zaifu-cho, Hirosaki 036-8562, Japan; nakaji@hirosaki-u.ac.jp; 3 Center for Advanced Medical Research, Department of Stress Response Science, Hirosaki University Graduate School of Medicine, 5 Zaifu-cho, Hirosaki 036-8562, Japan; ytatara@hirosaki-u.ac.jp; 4 Department of Social Health, Hirosaki University Graduate School of Medicine, 5 Zaifu-cho, Hirosaki 036-8562, Japan

**Keywords:** metabolomics, capillary electrophoresis–mass spectrometry, large-scale sampling, normalization, quality control, oxidative stress

## Abstract

For large-scale metabolomics, such as in cohort studies, normalization protocols using quality control (QC) samples have been established when using data from gas chromatography and liquid chromatography coupled to mass spectrometry. However, normalization protocols have not been established for capillary electrophoresis–mass spectrometry metabolomics. In this study, we performed metabolome analysis of 314 human plasma samples using capillary electrophoresis–mass spectrometry. QC samples were analyzed every 10 samples. The results of principal component analysis for the metabolome data from only the QC samples showed variations caused by capillary replacement in the first principal component score and linear variation with continuous measurement in the second principal component score. Correlation analysis between diagnostic blood tests and plasma metabolites normalized by the QC samples was performed for samples from 188 healthy subjects who participated in a Japanese population study. Five highly correlated pairs were identified, including two previously unidentified pairs in normal healthy subjects of blood urea nitrogen and guanidinosuccinic acid, and gamma-glutamyl transferase and cysteine glutathione disulfide. These results confirmed the validity of normalization protocols in capillary electrophoresis–mass spectrometry using large-scale metabolomics and comprehensive analysis.

## 1. Introduction

Many large-scale metabolomics studies have been performed for various purposes, such as prediction of the risk of developing diabetes [1,2], and evaluation of the associations between changes in specific groups of metabolites with antibiotic intervention and cardiovascular risk [3]. Recently, the Consortium of Metabolomics Studies [4] was established to promote collaboration and summarized 47 metabolomics cohort studies. Association of metabolite levels with the genome [5] and basic background information such as sex [6], age [7], and body mass index (BMI) [8] have also been reported. These studies provide useful information that could be used as a reference for analysis in clinical studies, such as biomarker discovery.

Recently, quality assurance and quality control (QA/QC) have been required in metabolomics. Variation during the performance of the metabolome analysis is generated in each step, including the sample handling process, wait time in the sampler before analysis, and data analysis. Han et al. [9] summarized the different types of variability in each step and pointed out the importance of strict adherence to standard operating procedures. Long et al. [10] have also divided the process into five steps and organized the pitfalls and practices in each step. It has been reported that there are three major problems during continuous sample measurement in large-scale metabolomics using gas chromatography (GC) and liquid chromatography (LC) coupled to mass spectrometry (MS): variation in the retention time, mass accuracy, and signal intensity [11,12]. For capillary electrophoresis–mass spectrometry (CE-MS) metabolomics, Drouin et al. [13] reported that high reproducibility could be achieved using the effective electrophoretic mobility instead of the relative migration time for identification of metabolites. High mass accuracy can be achieved by proper calibration during sample measurement.

Variation in the signal intensity or peak area in MS is caused by contamination of surfaces in continuous sample measurement, which causes drift in the measured response. To overcome this in large-scale metabolomics studies using LC-MS and GC-MS, it is recommended that quality control samples are analyzed along with each individual sample, and that the peak areas are normalized using the QC samples and smoothing approaches such as locally estimated scatterplot smoothing (loess) [11,12]. Harada et al. [14] reported that the coefficient of variation computed with QC samples using CE-MS data in a large-scale metabolomics cohort study was similar to or better than that with LC-MS and GC-MS data. However, the factors that affect the peak area during continuous sample measurement and the effectiveness of normalization using QC samples in CE-MS have not been fully investigated.

In this study, to determine which factors affect peak area variability in large-scale CE-MS, principal component analysis (PCA) was performed for metabolome data from QC samples. The peak areas for the QC samples were normalized using the smoothing trend computed by Whittaker smoothing [15]. Whittaker smoothing has the advantage that it can be computed easily, and it can compute smoothing estimates for samples after the last QC sample that other smoothing methods, such as loess, cannot compute. As an application to normalized metabolome data, partial correlation analysis was performed between diagnostic blood tests and plasma metabolites.

## 2. Results and Discussion

### 2.1. Exploration of Factors Causing Variation in Large-Scale Measurements

**Figure 1:**
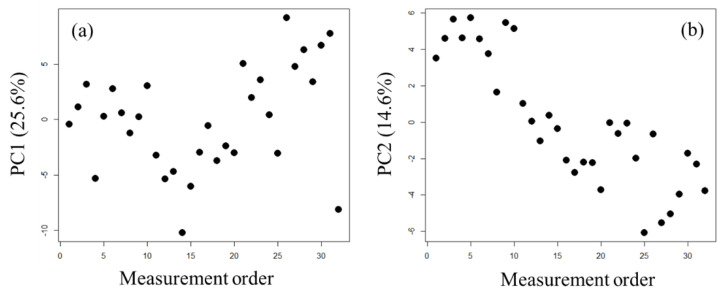
Results from PCA for QC samples for the PC1 score (**a**) and PC2 score (**b**).

In large-scale metabolome analysis using CE-MS, the factors that cause variation in peak areas during measurement have not been fully investigated. If we assume that the concentration of each metabolite in the QC samples is identical, variations during measurement can be considered as the source of most variability in the QC sample results. In this study, PCA was performed for the metabolomic data of the QC samples only (Figure 1), and the factors causing variation that were associated with the first or second principal component (PC1 and PC2, respectively) scores were examined.

With continuous measurement, the PC1 score showed a decrease and then an increase, and the PC2 score showed a constant decrease. We speculated that the variation in the PC1 score was because of the effect of capillary replacement. In practice, the capillary for the QC samples was replaced between sample numbers 5 and 6, 10 and 11, 20 and 21, and 23 and 24. The PC1 score for the peak area tended to decrease between the 10th and 11th QC samples and increase between the 20th and 21st QC samples. This result suggests that capillary exchange does not always affect the peak area. However, if the effect of capillary exchange appears in the peak area, then the effect is considered to be large. The PC2 score represented drift in the peak area [16,17] and showed a constant decrease with continuous measurement.

To select metabolites that were associated with the PC1 and PC2 scores, statistical hypothesis testing of PC loading [18] was performed (Table S1). Thirty one metabolites, including carnitine (*R* = −0.8890, *p* = 1.077 × 10^−11^, *q* = 9.266 × 10^−10^), tryptophan (*R* = −0.8828, *p* = 2.326 × 10^−11^, *q* = 1.977 × 10^−9^), and proline (*R* = −0.8676, *p* = 1.303 × 10^−10^, *q* = 1.094 × 10^−8^), were significantly correlated with the PC1 score (*q* < 0.05). All significant metabolites of PC1 loading were negative values. The PC1 scores first decreased and then increased, indicating that all significant metabolites in the PC1 loading had the opposite pattern, with an increase and then decrease. In PC2, sixteen metabolites, including uridine (*R* = 0.8141, *p* = 1.449 × 10^−8^, *q* = 1.246 × 10^−6^), guanidinoacetic acid (*R* = 0.7819, *p* = 1.262 × 10^−7^, *q* = 1.073 × 10^−5^), and 6-*N*-methyllysine (*R* = 0.7721, *p* = 2.280 × 10^−7^, *q* = 1.915 × 10^−5^), were significantly correlated with the PC2 score (*q* < 0.05). For these 16 significant metabolites, six negatively correlated metabolites, including choline (*R* = −0.7004, *p* = 8.071 × 10^−6^, *q* = 6.538 × 10^−4^), increased, and 10 positively correlated metabolites, including uridine, guanidinoacetic acid, and 6-*N*-methyllysine, decreased constantly with continuous measurement.

We used PCA to confirm that capillary replacement and a constant change with continuous measurement were the major variations. To reduce these variations, in addition to re-measuring the QC sample and the actual sample just before capillary replacement, like with LC-MS and GC-MS, it is useful in CE-MS to normalize the peak areas of the actual samples using the smoothing trend computed from the QC samples. All of the significantly correlated metabolites with PC1 scores were cationic metabolites. With the exception of mucic acid, threonic acid, and 3-phenylpropionic acid, all metabolites significantly correlated with the PC2 score were also cationic metabolites. These results suggest that variation during measurement is larger for cationic metabolites than for anionic metabolites.

The variation in peak area with replacement of the capillary is thought to occur because of slight differences in the position of the tip when the capillary is placed in the nebulizer, which introduces the sample from the CE device to the MS. As the capillary can break during measurement, capillary replacement is unavoidable. This problem could be solved in the future by development of an automated system to stably install the capillary in the nebulizer.

### 2.2. Evaluation of Normalization Using the QC Samples with Smoothing

In Section 2.1, we showed that metabolites such as carnitine, tryptophan, and proline had high values in the PC1 loading partially because of capillary replacement. Metabolites such as uridine, guanidinoacetic acid, and 6-*N*-methyllysine with high PC2 loading values varied constantly with continuous measurement. We attempted to normalize these variations with a smoothing trend computed using the QC samples, and the effect of this normalization was evaluated.

**Figure 2:**
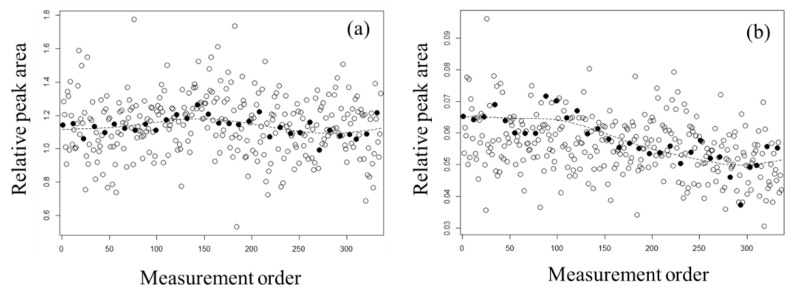
The smoothing trends for (**a**) carnitine and (**b**) uridine. Open circles indicate each individual’s actual samples and closed circles indicate QC samples. The dashed lines show estimated values of normalization using the QC samples with Whittaker smoothing.

To estimate the smoothing trend for normalization, we applied Whittaker smoothing to the peak areas of the QC samples. In doing so, the peak areas of the actual samples were treated as missing values. The smoothing parameter was set to a large value of κ = 10,000 in order to capture long-term rather than short-term trends. Figure 2 shows the results of smoothing for carnitine, which was the highest negatively correlated metabolite in the PC1 loading, and uridine, which was the highest positively correlated metabolite in the PC2 loading. For carnitine (Figure 2a), the smoothing curve was concave. The concavity of this curve was minor because the variability in the QC samples was relatively low compared with that in the actual samples for each individual. The smoothing curve of uridine (Figure 2b) decreased with continuous measurement. For comparison with other smoothing methods, the results calculated by loess are shown in Figure S1. The results of the Whittaker smoothing and loess were similar, except that the four samples measured after the last QC sample could not be calculated.

To confirm the effect of normalization with the smoothing trend on the QC samples, PCA was performed for the normalized data (Figure S2). The variations in the PC scores before normalization for capillary replacement and the constant change in the peak area (Figure 2) were not observed in the PC scores after normalization (Figure S2). To confirm the effect of normalization, correlation coefficients between the normalized peak areas and quantitative values were calculated by quantitative analysis using stable isotopes (Table S2). For lysine, the correlation coefficient improved slightly from 0.709 to 0.825 with normalization. For indole-3-acetic acid, the correlation coefficient improved significantly from 0.478 to 0.880. However, it should be noted that two samples were missing for indole-3-acetic acid and the correlation coefficient was computed using the remaining three samples. The metabolites alanine, glutamine, phenylalanine, valine, 2-oxoisovaleric acid, citric acid, lactic acid, and malic acid had high correlation coefficients of above 0.85 both before and after normalization. The relative standard deviations (RSDs) for peak areas before and after normalization with QC samples for each metabolite are shown in Table S3. The numbers of metabolites under 5%, between 5% and 10%, and over 10% RSD before normalization were 15, 38, and 33, respectively; the numbers of metabolites under 5%, between 5% and 10%, and over 10% RSD after normalization were 52, 27, and 7, respectively. The RSD was smaller after normalization than before normalization for all metabolites. These results suggest that normalization with smoothing using QC samples is effective.

### 2.3. Correlation Analysis between Plasma Metabolites and Diagnostic Blood Tests

**Figure 3:**
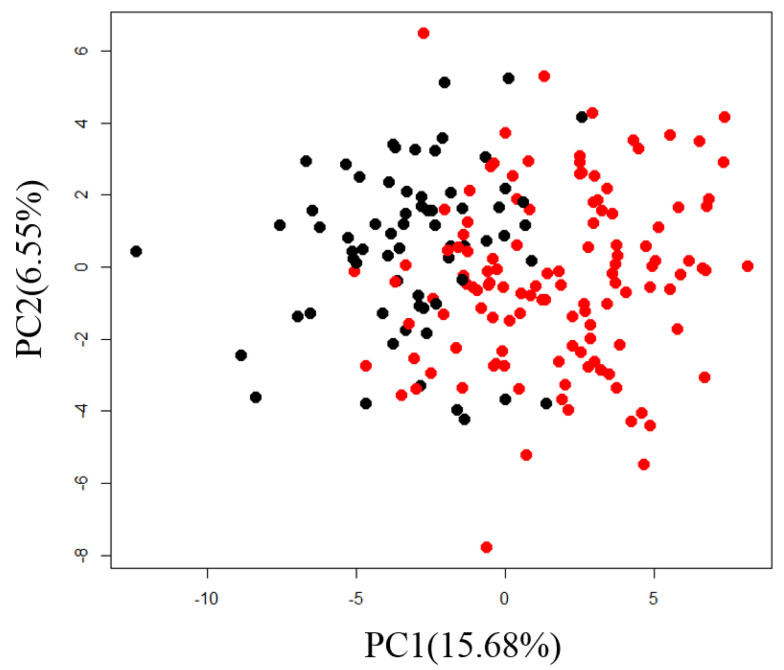
Scatter plot of PC1 and PC2 for normalized metabolome data from actual samples for individuals. Black circles are for samples from males and red circles are for samples from females.

Correlation analysis between the plasma metabolites and diagnostic blood tests was performed as an application of normalized metabolome data. First, we performed PCA and confirmed sex differences in PC1 (Figure 3).

**Figure 4:**
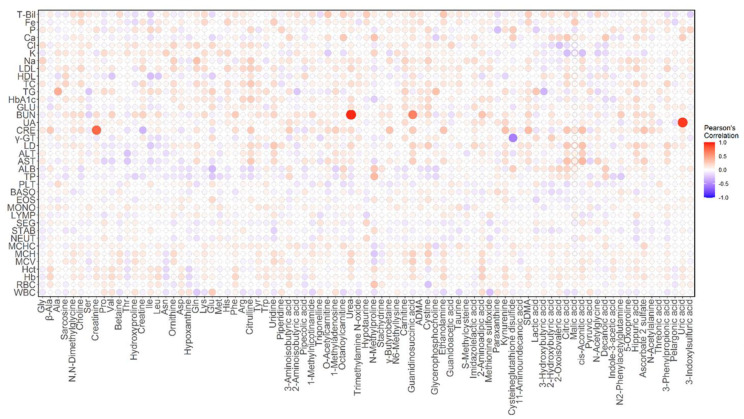
Heatmap of partial correlation coefficients between diagnostic blood tests and plasma metabolites. The colored bars gradually increase from white to blue for negative correlations and from white to red for positive correlations. The size of each spot changes according to the size of the correlation coefficient. Abbreviations in the figure are as follows: T-Bil: total bilirubin; Fe: serum iron; P: inorganic phosphorus; Ca: calcium; Cl: chlorine; K: potassium; Na: sodium; LDL: low-density lipoprotein cholesterol; HDL: high-density lipoprotein cholesterol; TC: total cholesterol; TG: triglycerides; HbA1c: hemoglobin A1c; GLU: glucose; BUN: blood urea nitrogen; UA: uric acid; CRE: creatinine; γ-GT: gamma-glutamyl transpeptidase; LD: lactate dehydrogenase; ALT: alanine transaminase; AST: aspartate aminotransferase; ALB: albumin; TP: total protein; PLT: platelet count; BASO: basophil; EOS: eosinophil; MONO: monocyte; LYMP: lymphocyte; SEG: segmented cell; STAB: stab cell; NEUT: neutrophil; MCHC: mean corpuscular hemoglobin concentration; MCH: mean corpuscular hemoglobin; MCV: mean corpuscular volume; Hct: hematocrit; Hb: hemoglobin; RBC: red blood cell count; WBC: white blood cell count.

Sixty-one metabolites, or more than 70% of the total number of analyzed metabolites, were significantly correlated with the PC1 scores (*q* < 0.05, Table S3). This result suggests that many metabolites differed by sex, so we calculated partial correlation coefficients with sex as a confounding variable. A heatmap of the correlation between diagnostic blood tests and plasma metabolites is shown in Figure 4.

**Figure 5:**
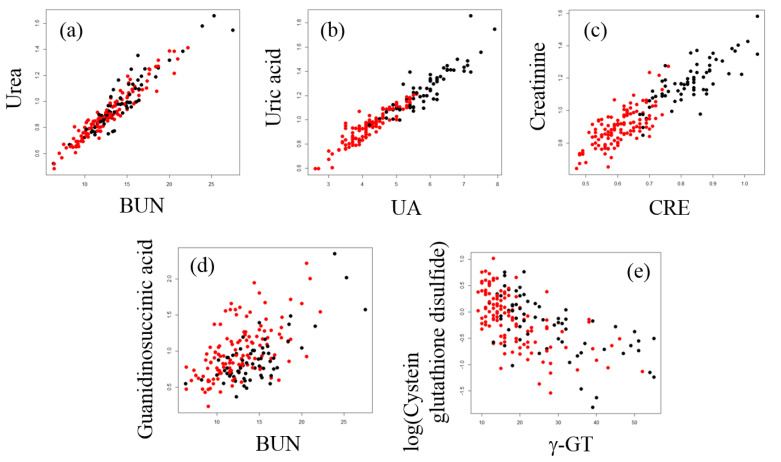
Scatter plot of five pairs of diagnostic blood tests and plasma metabolites. Black circles indicate samples from males and red circles indicate samples from females. (**a**) Blood urea nitrogen (BUN) test and urea, (**b**) uric acid (UA) test and uric acid, (**c**) creatinine (CRE) test and creatinine, (**d**) BUN test and guanidinosuccinic acid, and (**e**) gamma-glutamyl transpeptidase (γ-GT) test and cysteine glutathione disulfide.

Among all combinations of partial correlation coefficients between the diagnostic blood tests and metabolites, the top five pairs with the highest correlation coefficients were as follows: blood urea nitrogen (BUN) and urea (*R* = 0.9347, *p* = 4.583 × 10^−85^; Figure 5a), uric acid (UA) and uric acid (*R* = 0.8999, *p* = 1.347 × 10^−68^; Figure 5b), creatinine (CRE) and creatinine (*R* = 0.7466, *p* = 1.410 × 10^−34^; Figure 5c), BUN and guanidinosuccinic acid (GSA) (*R* = 0.6053, *p* = 4.433 × 10^−20^; Figure 5d), and gamma-glutamyl transpeptidase (γ-GT) and cysteine glutathione disulfide (CSSG) (*R* = −0.5649, *p* = 3.716 × 10^−17^). The partial correlation coefficient between γ-GT and the log-transformed data of CSSG was slightly better than that before the transformation (*R* = −0.6176, *p* = 4.712 × 10^−21^; Figure 5e). It is reasonable that the partial correlation coefficients between BUN and urea, UA and uric acid, and CRE and creatinine were close to one because we were measuring the same substance although the sample species and the measurement methods were different.

**Figure 6:**
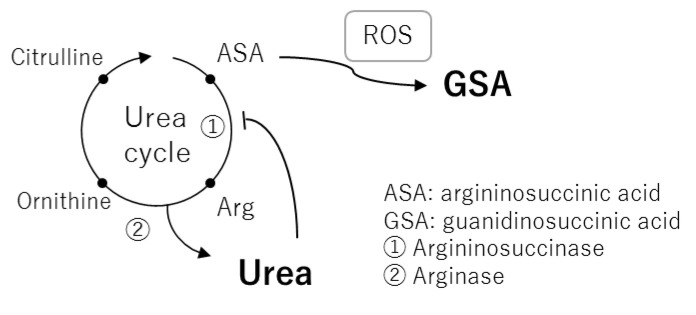
A metabolic pathway map of the urea cycle and related metabolites as a basis for correlation between blood urea nitrogen (BUN) and guanidinosuccinic acid.

In the current study, we showed for the first time that GSA was correlated with BUN in normal healthy subjects (Figure 5d). In 1977, GSA was detected in blood and urine samples from uremic patients and correlated with BUN [19]. This correlation is normally explained by formation of GSA through the guanidine cycle from excess urea [20]. However, it is apparent that GSA is also produced by aberrant cleavage of argininosuccinic acid, an intermediate of the urea cycle, by reactive oxygen species such as hydroxy radicals [21,22]. The association of GSA with BUN is reasonable because their generation may commonly depend on the flux of the urea cycle and urea inhibition of argininosuccinase (Figure 6) [23]. Aoyagi et al. demonstrated that urea strongly increased the generation of GSA in rat primary hepatocytes and that norvaline, an inhibitor of the urea cycle, markedly decreased urea-induced generation of GSA [24]. However, generation of GSA from argininosuccinic acid probably only occurs under oxidative stress because it does not appear in rare cases of argininosuccinase deficiency. We speculated that two factors, one that leads to the increased flux of urea cycle, such as an increase in protein catabolism, and the other being oxidative stress, coordinate to contribute to the synthesis of GSA. Thus, GSA could be a marker of oxidative stress when it is affected by the flux of the urea cycle.

**Figure 7:**
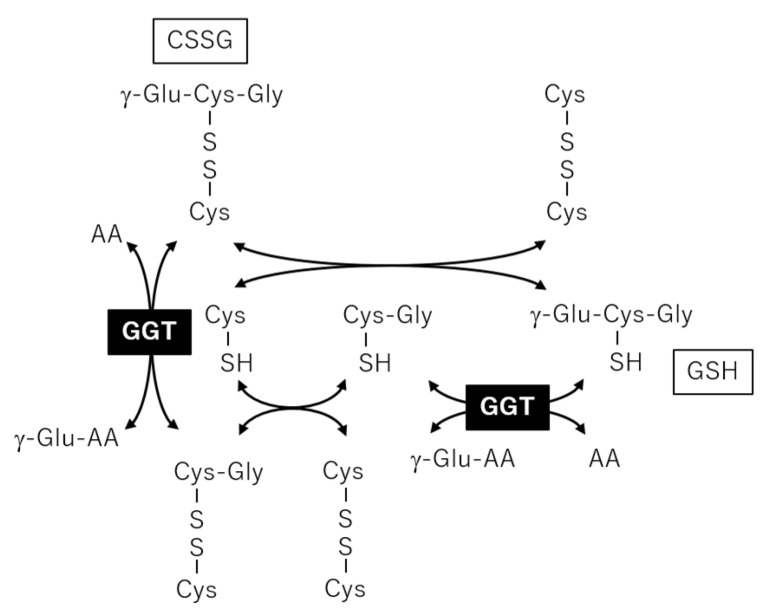
Metabolic reactions among glutathione and cysteine glutathione disulfide (CSSG) to provide a basis for correlation between gamma-glutamyl transpeptidase (γ-GT) and CSSG.

We also found a correlation between γ-GT and CSSG. Usually, quantification of oxidatively labile thiols, such as GSH and cysteines, in cohort plasma samples is difficult because in vitro oxidation and thiol exchange reactions occur during sampling and analysis. Consequently, GSH and cysteine concentrations are usually under the detection limit, which occurred in this study. In a previous report, plasma thiols were detected using alkylating reagents to avoid artificial reactions in vitro, and the GSH concentration was correlated with CSSG [25]. Jones et al. argued that CSSG was generated in vivo from the reaction of GSH with cystine, which is present in relatively high concentrations in human plasma. Furthermore, GSH added to human plasma in vitro rapidly undergoes a thiol-exchange reaction with cystine, leading to artificial generation of CSSG [26]. Thus, the CSSG measured in our analysis should be the sum of endogenous CSSG plus that artificially generated in vitro, both of which are derived from the thiol-exchange reaction of GSH and cystine. γ-GT is an enzyme that plays a key role in the gamma-glutamyl cycle, a pathway for the synthesis and degradation of GSH, and catalyzes the transfer of gamma-glutamyl groups of GSH to an acceptor [27]. Plasma or membrane bound γ-GT may affect the GSH metabolism both in vivo and in vitro, and the negative correlation between CSSG and γ-GT shown in the present study (Figure 5e) may reflect the negative effect of γ-GT on CSSG generation (Figure 7).

## 3. Materials and Methods

### 3.1. Human Participants and Sample Collection

Plasma samples were collected from subjects recruited in the Iwaki Health Promotion Project, which is a health promotion study of Japanese people over 20 years of age that aims to prevent lifestyle-related diseases and prolong lifespans. The study protocol was approved by the Ethics Committee of the Hirosaki University Graduate School of Medicine (Hirosaki, Japan), and written informed consent was obtained from all participants. There were 3137 subjects enrolled in the Iwaki project in 2016 and 2017, and some of these subjects were excluded from our study in the same manner as in our previous study [28]. The criteria for exclusion of the subjects are described in Section 3.2. The only difference compared with the previous study is that we used CRE instead of creatine kinase because creatine kinase was not measured in this study. After applying the exclusion criteria, 314 samples remained and metabolome analysis was performed for these samples. Among these samples, some were from the same individual but collected in different years (i.e., 2016 and 2017). In these cases, the samples from 2016 was selected for the analysis. In total, 314 samples obtained from 188 healthy subjects were used for the statistical analysis. The demographic data of 188 healthy subjects are shown in Table 1.

### 3.2. Exclusion Criteria for Medical Parameters and Diagnostic Blood Tests

The medical parameters used to exclude samples from certain individuals are described below. First, individuals that were taking prescription medications at the time of sampling were excluded. Next, individuals with samples that had results within the limits shown in Table 2 were excluded. Finally, we excluded individuals that smoked more than 20 cigarettes per day, had a BMI of ≤14 kg/m^2^ or ≥30 kg/m^2^, or had average blood pressure of ≥160 mmHg in the systolic phase and ≥100 mmHg in the diastolic phase.

The diagnostic blood tests used for correlation analysis were the white blood cell count, red blood cell count, hemoglobin (Hb), hematocrit (Hct), mean corpuscular volume (MCV), mean corpuscular hemoglobin, mean corpuscular hemoglobin concentration, neutrophil, stab cells, segmented cells, lymphocytes, monocytes, eosinophil, basophil, platelet count, total protein, albumin (ALB), aspartate aminotransferase, alanine transaminase, lactate dehydrogenase, gamma-glutamyl transpeptidase (γ-GT), creatinine (CRE), uric acid (UA), BUN, glucose (GLU), Hb A1c, triglycerides (TG), total cholesterol, high-density lipoprotein cholesterol, low-density lipoprotein cholesterol, sodium (Na), potassium (K), chlorine (Cl), calcium (Ca), inorganic phosphorus (P), serum iron (Fe), and total bilirubin.

### 3.3. Metabolome Analysis

Metabolome analysis was conducted at Human Metabolome Technologies (HMT; Tsuruoka, Japan). Briefly, 50 µL of plasma was added to 450 µL of methanol containing internal standards (solution ID: H3304-1002, HMT) at 0 °C to inactivate enzymes. The solution was thoroughly mixed with 500 µL of chloroform and 200 µL of Milli-Q water and centrifuged at 2300× *g* and 4 °C for 5 min. Then, 350 µL of the upper aqueous layer was centrifugally filtered through a Millipore 5-kDa cutoff filter to remove proteins. The filtrate was centrifugally concentrated and re-suspended in 50 µL of Milli-Q water for metabolome analysis at HMT.

Metabolome analysis was performed by CE coupled with time-of-flight (TOF)-MS [29]. Electrolytes, the sheath liquid, authentic materials for migration-time correction, and internal standards were all prepared from a HMT metabolomics kit. Standard mixtures for metabolite annotation and quantification were prepared using 403 commercially available compounds. Methanol was purchased from Kanto Chemical Co., Inc. (Tokyo, Japan). Chloroform was purchased from FUJIFILM Wako Pure Chemical Corporation (Osaka, Japan).

CE-TOF-MS was carried out using a 7100 CE System, 6210 TOF-MS, 1100 isocratic high-performance liquid chromatography pump, G1603A CE-MS adapter kit, and G1607A CE-ESI-MS sprayer kit, all from Agilent Technologies Inc. (Santa Clara, CA, USA). The systems were controlled by ChemStation software (version B.04.02 SP1) and MassHunter Data Acquisition for TOF/Q-TOF (version B.02.00, Agilent Technologies Inc.). The sheath flow rate was set at 10 µL/min. Metabolites were separated using a fused silica capillary (50 µm i.d. × 80 cm total length; Polymicro Technologies, Inc., Phoenix, AZ, USA) with 50 mM ammonium acetate (pH 8.5) for anion analysis and 1 M formic acid for cation analysis. The applied voltage for CE was set at 30 kV. MS spectra were scanned from *m/z* 50 to 1000. The TOF-MS acquisition rate was set at 1.5 spectra/s. MS was conducted in negative ionization mode with 3500 V for anion analysis and in positive ionization mode with 4000 V for cation analysis.

Data processing for peak picking, peak alignment, metabolite annotation, and peak integration was performed using MasterHands™, developed by the Institute for Advanced Biosciences, Keio University [30]. For metabolome analysis, the migration time of each peak detected by CE-MS was corrected using those of the internal standards and authentic materials for migration-time correction. Putative metabolites were identified according to the *m/z* and corrected migration times acquired from a standard mixture that was analyzed using the same CE conditions. The tolerance was ±10 ppm for the *m/z* and ±0.5 min for the corrected migration time. The results were checked manually, then the peak area of each metabolite was normalized to that of the appropriate internal standard. After this, 86 metabolites remained and this dataset was used for statistical analysis.

Quantitative analysis using ^13^C was also performed for five randomly selected samples. Quantitative values for alanine, glutamate, lysine, phenylalanine, valine, 2-oxoisovaleric acid, citric acid, indole-3-acetic acid, lactic acid, and malic acid were computed.

### 3.4. QC Samples and Normalization with Whittaker Smoothing

**Figure 8:**
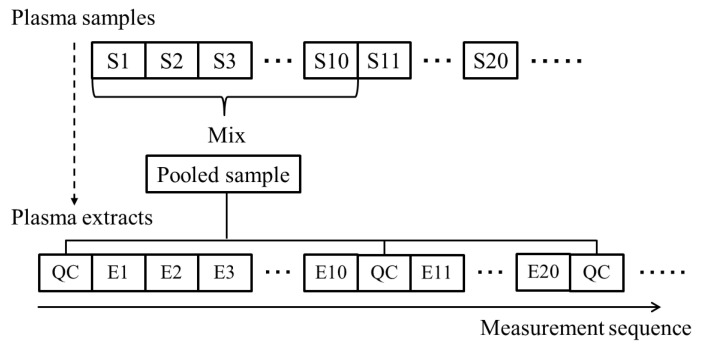
Overview of the procedure for preparation of pooled quality control (QC) samples and the measurement sequence for all samples, including the actual samples for each individual and QC samples. The letter *“*S*”* stands for samples and *“*E*”* for extracts.

QC samples were prepared by mixing 100 µL aliquots of 10 samples randomly selected from the full sample set (*n* = 314). The QC samples were analyzed after every 10 samples (Figure 8). We calculated the smoothing curve using the peak areas of the QC samples, and the peak areas of the individual plasma samples were normalized using a smoothing trend computed by Whittaker smoothing.

We used the version of Whittaker smoothing introduced by Eilers [15]. We set the peak area of the QC sample as **y** and the estimated smoothed value as **z**. Whittaker smoothing is defined as minimizing *E* in the following equation:

(1) $$E=\sum_{i}w_{i}{\left(y_{i}-z_{i}\right)}^{2}+\lambda \sum_{i}Dz_{i}{}^{2}={\left(y-z\right)}^{\prime }W\left(y-z\right)+\lambda z^{\prime }D^{\prime }Dz,$$

where *i* is the *i*-th sample; *λ* is a parameter that represents the degree of smoothing; **D** is the differential matrix; and **W** is a diagonal matrix with a diagonal component, in which the QC sample corresponds to one and each individual’s actual samples to zero. To perform smoothing using the QC samples and estimate each individual’s actual sample for normalization, the matrix **W** is necessary to treat non-QC samples (i.e., each individual’s samples) as missing values.

The first term in Equation (1) is the weighted squared error of the data and the estimate from smoothing, and the second term represents the degree of smoothing. We performed partial differentiation of **z** and set it to 0, which can be written as follows:

(2) $$\left(W+\lambda D_{d}^{\prime }D_{d}\right)z=Wy.$$

The value of **z** can be estimated by multiplying the inverse matrix of $W+\lambda D_{d}^{\prime }D_{d}$ from Equation (2) as follows:

(3) $$z={\left(W+\lambda D_{d}^{\prime }D_{d}\right)}^{-1}Wy.$$

A normalized value can be calculated by dividing the peak area for an individual’s actual sample by the estimated value **z**. We used the second differential matrix **D_2_** as the differential matrix, which can be written as follows:

(4) $$D_{2}=\left[\begin{array}{cccccccc} -1 & 2 & 1 & 0 & \cdots & 0 & 0 & 0\\ 0 & -1 & 2 & 1 & \cdots & 0 & 0 & 0\\ \vdots & \vdots & \vdots & \vdots & \ddots & \vdots & \vdots & \vdots \\ 0 & 0 & 0 & 0 & \cdots & 1 & -2 & 1 \end{array}\right].$$

### 3.5. Statistical Analysis

All statistical analysis was performed using R software ver. 3.6.0. Whittaker smoothing was computed using an in-house R program. Partial correlation coefficients were calculated using the ppcor package ver. 1.1, and the correlation matrix was drawn using the ggcorrplot package ver. 0.1.3.

## 4. Conclusions

We analyzed the factors causing variations in large-scale CE-MS metabolomics and found that capillary replacement and a linear trend with continuous measurement were major factors. To reduce these variations, the peak areas of the actual samples were normalized using those of QC samples. Analysis of QC samples before and after normalization and comparison with quantitative values using stable isotopes confirmed that metabolome data quality was improved by normalization. As an application of the normalized metabolome data, we performed correlation analysis between diagnostic blood tests and plasma metabolites for samples from healthy Japanese subjects. We found two pairs, BUN and GSA, and γ-GT and CSSG, that have previously not been identified in normal healthy subjects. These results confirmed the validity of normalization protocols in CE-MS using large-scale metabolomics and comprehensive analysis.

## Figures and Tables

**Table 1:** Demographic data of 188 healthy subjects.

	Male	Female	Total
Sample size	66	122	188
Age (year)			
Mean	42.9	45.1	44.3
Median	39.5	43.5	43
Range	20–74	20–73	20–74
BMI (kg/m^2^)			
Mean	23.1	21.2	21.9
Median	22.85	21.1	21.5
Range	18.2–29	15.5–28.1	15.5–29

BMI: body mass index.

**Table 2:** The exclusion criteria of the blood tests.

Blood Tests	Lower Limit	Upper Limit
ALB (g/L)	<41	>51
TG (mmol/L)	<0.47 (M), <0.34 (F)	>2.51 (M), >1.4 (F)
UA (mmol/L)	<224 (M), <154 (F)	>474 (M), >334 (F)
GLU (mmol/L)	<4.2	>5.9
γ-GT (U/L)	<9	>55
CRE (mmol/L)	<0.64 (M), <0.46 (F)	>1.06 (M), >0.78 (F),
C-reactive protein (mg/L)	-	>1.4
Hb (g/L)	<135 (M), <110 (F)	>169 (M), >148 (F)
MCV (fl)	<82	>98

ALB: albumin; TG: triglycerides; UA: uric acid; GLU: glucose; γ-GT: gamma-glutamyl transpeptidase; CRE: creatinine; Hb: hemoglobin; MCV: mean corpuscular volume; M: male; F: female.

## Data Availability

The data presented in this study are available under the agreement with Hirosaki University Center of Healthy Aging Innovation.

## Supplementary Materials

The following are available online at https://www.mdpi.com/article/10.3390/metabo11050314/s1: Table S1: Statistical significantly metabolites correlated with PC1 and PC2 scores in the metabolome data for the QC samples, Table S2: Correlation coefficients between before and after normalization and quantitative values from quantitative analysis using stable isotopes, Table S3: The relative standard deviation (RSD) of the peak area before and after normalization with QC samples for each metabolite, Table S4: Statistically significantly metabolites correlated with the PC1 score in normalized metabolome data, Figure S1: The smoothing trends for (a) carnitine and (b) uridine. Open circles indicate each individual’s actual samples and closed circles indicate QC samples. The dashed lines show estimated values of normalization using the QC samples with locally estimated scatterplot smoothing (loess), Figure S2: Results of PCA for normalized metabolome data from QC samples. The vertical axes show the PC1 score (a) and PC2 score (b).
