# Supplementary material for: Capillary Electrophoresis Mass Spectrometry-Based Metabolomics of Plasma Samples from Healthy Subjects in a Cross-Sectional Japanese Population Study
Source: Metabolites. 2021 May 13;11(5):314. doi: 10.3390/metabo11050314 (PMC8153282; doi:10.3390/metabo11050314)
Supplement: Supplementary file 1 [file metabolites-11-00314-s001.zip › Supplementary_Figure.pdf]

Supplementary Figures

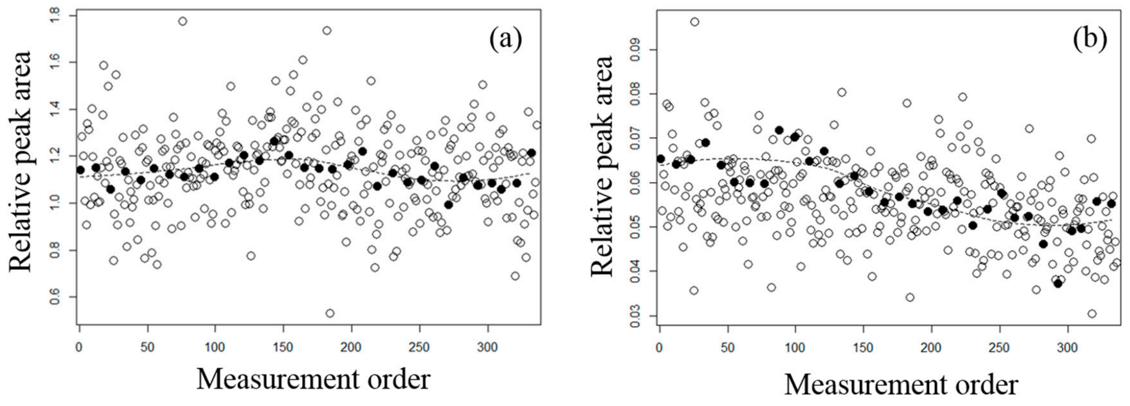

Figure S1: The smoothing trends for (a) carnitine and (b) uridine. Open circles indicate each individual's actual samples and closed circles indicate QC samples. The dashed lines show estimated values of normalization using the QC samples with locally estimated scatterplot smoothing (loess).

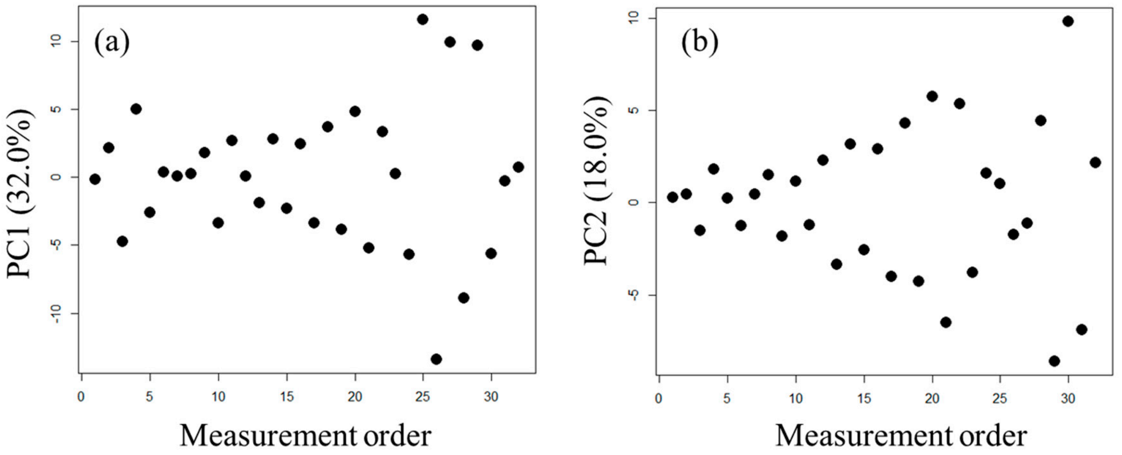

Figure S2: Results of PCA for normalized metabolome data from QC samples. The vertical axes show the PC1 score (a) and PC2 score (b).
