# Supplementary figures and images for: Capillary Electrophoresis Mass Spectrometry-Based Metabolomics of Plasma Samples from Healthy Subjects in a Cross-Sectional Japanese Population Study
Source: Metabolites. 2021 May 13;11(5):314. doi: 10.3390/metabo11050314 (PMC8153282; doi:10.3390/metabo11050314)

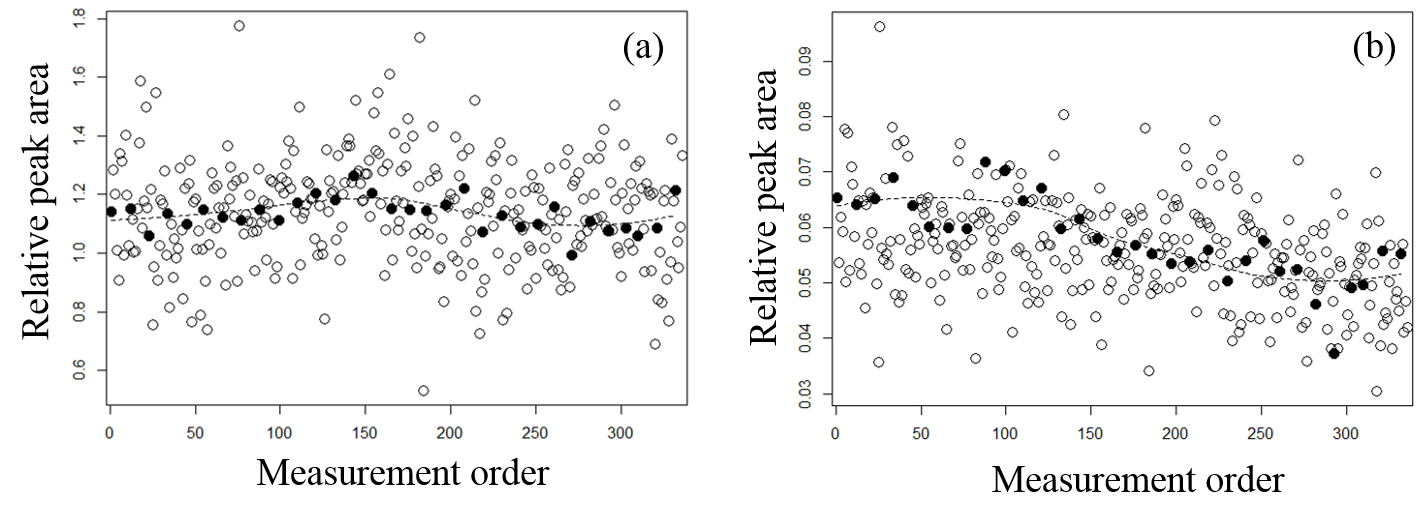

Supplement: Supplementary file 1 [file metabolites-11-00314-s001.zip › FIGS1.png]

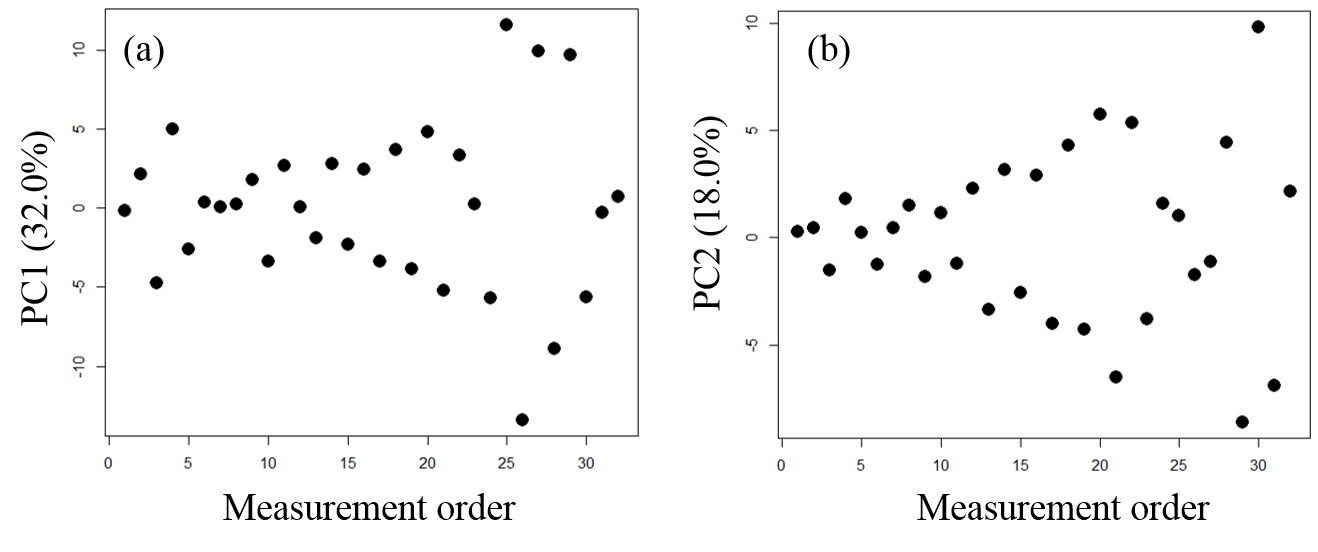

Supplement: Supplementary file 1 [file metabolites-11-00314-s001.zip › FIGS2.png]
